# Sleep-Disordered Breathing and Cardiac Arrhythmias

**DOI:** 10.3390/jcm13226635

**Published:** 2024-11-05

**Authors:** Tushar Menon, Ikechukwu Ogbu, Dinesh K. Kalra

**Affiliations:** Division of Cardiology, Department of Medicine, University of Louisville School of Medicine, Louisville, KY 40202, USA; t0meno02@louisville.edu (T.M.); i0ogbu01@louisville.edu (I.O.)

**Keywords:** obstructive sleep apnea (OSA), central sleep apnea (CSA), sleep-disordered breathing (SDB), atrial fibrillation, ventricular arrhythmias (VA), sudden cardiac death (SCD), bradyarrhythmia, continuous positive airway pressure (CPAP)

## Abstract

A narrative review was performed, analyzing peer-reviewed articles from databases such as PubMed, EMBASE, Scopus, and Web of Science to examine the mechanistic links between sleep-disordered breathing (SDB), and cardiac arrhythmias, emphasizing intermittent hypoxia, autonomic imbalance, and intrathoracic pressure swings as key pathways. Studies, including the DREAM and CESAAR trials, consistently demonstrate that SDB patients face elevated risks: more than doubling the likelihood of overall arrhythmias (OR 2.24; 95% CI 1.48–3.39), quadrupling the risk of AF (OR 4.02; 95% CI 1.03–15.74), and tripling the risk of non-sustained ventricular tachycardia (NSVT) with higher apnea-hypopnea index (AHI) values. Additionally, SDB doubles the risk of bradyarrhythmia, such as sinus pause, second and third-degree atrioventricular block, and intraventricular conduction delay (OR 2.50; 95% CI 1.58–3.95). According to meta-analytical findings, continuous positive airway pressure (CPAP) therapy is a pivotal intervention, significantly reducing AF recurrence by 42% and lowering VA incidence by 58%. Moreover, CPAP therapy diminishes sinus bradycardia and occurrences of sinus pause and may reduce the necessity for pacemaker implantation. Recognizing SDB as a modifiable risk factor for cardiac arrhythmias highlights the importance of early diagnosis and effective management, primarily through CPAP therapy, to improve cardiovascular outcomes.

## 1. An Overview of Obstructive Sleep Apnea and Central Sleep Apnea

Obstructive sleep apnea (OSA), affecting nearly a billion people globally, is marked by recurrent upper airway collapse during sleep, causing intermittent hypoxemia, hypercapnia, frequent arousals, and fragmented sleep. These disruptions alter sleep architecture and trigger neurohormonal, cardiovascular, and metabolic changes, elevating the risk of heart disease, metabolic dysfunction, and neurocognitive impairment. There are various mechanisms (endotypes) and clinical manifestations (phenotypes) of OSA, each influencing the disease’s severity, treatment response, and prognosis differently [1,2]. Phenotypes range from asymptomatic individuals to those with significant sleep disruption and daytime sleepiness, with associated varying cardiovascular risks [1,2].

Central sleep apnea (CSA), often linked to chronic heart failure (HF), stroke, or opioid use, is marked by reduced ventilatory effort and decreased airflow. CSA has various subtypes driven by an elevated loop gain, ventilatory instability, neuromuscular dysfunction, and low arousal threshold [3,4]. Elevated loop gain refers to an increased sensitivity to ventilation disturbances, where minor blood gas changes trigger cycles of hyperventilation and apnea, and is common in HF [3,4]. This ventilatory instability leads to irregular cycles of hyperventilation and apnea due to heightened carbon dioxide sensitivity [3,4]. Neuromuscular dysfunction impairs the ventilatory drive by weakening the respiratory muscles, leading to subsequent hypoventilation [3,4]. Additionally, CSA can be induced or worsened by continuous positive airway pressure (CPAP) therapy—while CPAP is a primary treatment for OSA, it can paradoxically worsen apnea events in CSA patients by reducing the CO_2_ levels below the apneic threshold, reducing the ventilatory drive [3,4].

Both obstructive sleep apnea (OSA) and central sleep apnea (CSA), collectively known as sleep-disordered breathing (SDB), are significantly associated with an increased incidence of arrhythmias. This association is attributed to various pathophysiological mechanisms, including repetitive hypoxia and subsequent reoxygenation, which lead to inflammation and oxidative stress. Additionally, these processes activate the sympathetic nervous system, contributing to cardiac remodeling, interstitial fibrosis, ion channel disturbances, anisotropic conduction, and the development of cardiac arrhythmias.

## 2. Epidemiological Studies Linking Sleep-Disordered Breathing to Cardiac Arrhythmias

Several studies have reported on the epidemiological link between SDB and arrhythmias. The Sleep Heart Health Study (SHHS) and the Determining Risk of Vascular Events by Apnea Monitoring (DREAM) studies showed an increased prevalence of atrial fibrillation (AF), ventricular arrhythmias (VA), and bradyarrhythmias among individuals with SDB.

The DREAM study examined the relationship between SDB and nocturnal cardiac arrhythmias in 1011 clinic-based patients. Individuals with moderate to severe SDB had twice the risk of nocturnal arrhythmias, including complex ventricular and supraventricular tachycardias, compared to those without SDB (OR 2.24; 95% CI, 1.48–3.39), as well as bradyarrhythmias such as sinus pause and atrioventricular block (OR: 2.50; *p* = 0.001) [5,6]. The oxygen desaturation index (ODI) emerged as a potent predictor of arrhythmias, highlighting hypoxia’s role in arrhythmogenesis [5,6]. A meta-analysis involving 4852 patients found that the prevalence of bradycardia was 25% (95% CI: 18.6–32.7) during the day and 69.8% (95% CI: 41.7–88.2) at night [7]. Notably, obstructive respiratory events were stronger predictors of arrhythmias than central events due to their more pronounced effects on cardiac pressure dynamics.

The Sleep Heart Health Study (SHHS), a multicenter longitudinal study of over 6000 U.S. participants, showed that individuals with moderate to severe SDB (AHI ≥ 30) compared to those with mild SDB (AHI ≤ 5) had high rates of atrial fibrillation (4.8% vs. 0.9%; *p* = 0.003), non-sustained ventricular tachycardias (5.3% vs. 1.2%; *p* = 0.004), and complex ventricular ectopy (25% vs. 14.5%; *p* = 0.002) [8,9,10]. Though bradyarrhythmias were more frequent in SDB patients, differences were not statistically significant. These results highlight the need for targeted arrhythmia screening in SDB (Table 1).

## 3. Epidemiological Evidence of Sleep Apnea as a Risk Factor for Atrial Fibrillation

Linz et al. showed that in a cohort of 1000 patients, there was a significant difference in OSA prevalence: 21% to 74% in AF patients versus 3% to 49% in controls [8]. This disparity emphasizes the close relationship between OSA and AF, highlighting shared pathophysiological mechanisms. The variability in prevalence reflects differences in diagnostic criteria, ranging from subjective questionnaires like the Epworth Sleepiness Scale to objective measures such as polysomnography (PSG) and portable sleep studies assessing the AHI. Despite methodological differences, these studies consistently show an increased prevalence of OSA in AF patients.

Expanding on the complexities of OSA’s impact on AF, The Sleep Signals, Testing, and Reports Linked to Patient Traits (STARLIT) registry’s retrospective cohort study explored how sleep phenotypes influence AF risk. This study analyzed in-lab PSG data from 43,433 patients, linking sleep traits to AF incidence over an average follow-up of 7.6 years. Findings revealed significant variations in AF risk across phenotypes: the hypoxia phenotype was associated with 48% increased risk (HR: 1.48), apnea plus arousal phenotype was associated with 22% increased risk (HR: 1.22), and short sleep plus non-REM showed an 11% increased risk [9]. This was further supported by the Health eHeart and Cardiovascular Health Study, which showed that frequent nighttime awakenings significantly increased the risk of AF by 33% over a median follow-up period of 11.6 years (HR: 1.33; *p* < 0.001). Additionally, a reduction in rapid eye movement (REM) sleep was associated with a higher risk of AF, with each standard deviation decrease in REM sleep increasing AF risk by 18% among certain participants (HR 1.18; *p* = 0.047) [10].

The study “Association of Short Sleep Duration and Atrial Fibrillation” examined the link between sleep duration and AF across a large cohort of 31,079 patients who underwent diagnostic polysomnography. This investigation revealed that for every one-hour reduction in total sleep time, there was a 17% increase in the risk of prevalent AF and a 9% increase in the risk of incident AF [12]. Notably, the study reported that the patients who slept less than three hours had 2.10 times higher odds of prevalent AF than those who slept more than six hours [12,21]. Additionally, the longitudinal analysis showed that shorter sleep duration significantly decreased AF-free survival over a median follow-up of 4.6 years [12,21]. This illustrates the public health importance of adequate sleep in managing and potentially reducing the risk of atrial fibrillation.

The “Healthy Sleep Patterns and Risk of Incident Arrhythmias” reveals the interplay between sleep quality and cardiovascular health using a substantial cohort of 403,187 participants from the UK Biobank. This study used an integrative score encompassing chronotype, sleep duration, insomnia, snoring, and daytime sleepiness to correlate sleep quality with the risk of atrial fibrillation/flutter. Participants with the highest adherence to healthy sleep behaviors (a sleep score of 5) demonstrated a 29% reduction in the risk of developing atrial fibrillation and a 35% reduction in bradyarrhythmia compared to those with poor sleep patterns (a sleep score of 0–1) [13]. Additionally, optimal sleep duration (7–8 h per night) and the absence of frequent insomnia were associated with significant reductions in the risk of atrial fibrillation, with hazard ratios of 0.92 and 0.88, respectively [13]. This highlights the importance of managing sleep quality as a modifiable risk factor in cardiovascular health strategies.

In the I-STOP-AFib trial, an innovative n-of-1 randomized clinical study, participants with paroxysmal AF were empowered to test self-selected triggers, including reduced sleep, to determine their potential impact on AF episodes. This trial involved 446 participants and allowed individuals to actively manage their health by tracking potential AF triggers through a mobile application [14,15]. Notably, reduced sleep was selected by 31 participants as a suspected trigger, reflecting its perceived relevance in AF pathophysiology [14,15]. The trial’s results, however, did not conclusively link reduced sleep to an increased risk of AF episodes, as evidenced by the lack of significant differences in the Atrial Fibrillation Effect on Quality-of-Life (AFEQT) scores between the n-of-1 and control groups [14,15]. The study’s findings underscore the complex and individualized nature of AF triggers and highlight the potential disconnect between perceived triggers and their verifiable impact on AF events.

The VARIOSA-AF observational cohort study greatly enhanced our understanding of the temporal dynamics between SDB and AF. Utilizing implanted monitoring devices capable of daily assessments, this study tracked the respiratory disturbance index (RDI) and AF burden in a cohort of 72 patients over a prolonged follow-up period [11]. Analysis revealed on nights where the highest quartile of RDI was recorded (indicating severe SDB), there was a statistically significant increase in the risk of AF episodes the following day, with odds ratios ranging from 1.7 for AF episodes lasting more than 5 min to an astonishing 10.2 for episodes exceeding 12 h [11].

## 4. Epidemiological Evidence of Sleep Apnea as a Risk Factor for Ventricular Arrhythmias and Sudden Cardiac Death

SDB has been strongly associated with a higher incidence of VA and SCD, especially during nighttime. Recent studies provide compelling evidence that the severity of SDB correlates with an increased occurrence of these life-threatening arrhythmias.

In a longitudinal cohort study involving 10,701 adults, researchers investigated the link between OSA and the elevated risks of SCD. The study found that individuals with an AHI > 20 and nocturnal oxygen saturations below 78% had a significantly increased risk of SCD (hr: 1.60 (*p* = 0.0007) and 1.81 (*p* = 0.00008), respectively) [16]. Additionally, data were published in The New England Journal of Medicine from a detailed analysis of polysomnograms and death certificates of 112 subjects who experienced SCD from midnight to 6 a.m., a time typically associated with the lowest risk in the general population and among individuals without OSA [18]. This demonstrated that 46% of those with OSA died from cardiac causes during these early morning hours, significantly higher than the 21% observed in individuals without OSA (*p* = 0.01) and substantially exceeding the 16% expected in the general population (*p* < 0.001) [18,22]. Further analysis revealed that the relative risk of SCD during this period was 2.57 for those with OSA [18]. These individuals also exhibited a significantly higher median AHI than those who died during other times of the day.

The “Central Sleep Apnea and Arrhythmogenesis After Myocardial Infarction (CESAAR) study” further emphasized the relationship between SDB and ventricular arrhythmias by looking at the relationship between SDB and the incidence of NSVT during the subacute recovery phase of acute myocardial infarction (AMI) [17]. CSA was diagnosed in 44.9% of the 202 subjects with SDB, and of those who had an AHI > 23, there was an associated threefold increase in the risk of NSVT (OR: 3.7; 9), highlighting the detrimental effects of nocturnal hypoxemia [17].

In conclusion, the increasing body of research substantiates the significant role of SDB in the epidemiology of VA and SCD, particularly during nocturnal hours when the risk should be lowest. These findings highlight the necessity for enhanced screening and targeted interventions in individuals diagnosed with SDB to mitigate these severe cardiac risks.

## 5. Epidemiological Evidence of Sleep Apnea as a Risk Factor for Bradyarrhythmia

SDB has been linked to a higher incidence of bradyarrhythmias due to increased vagal activity, which may occur during periods of oxygen desaturation [5,6,23,24]. While bradyarrhythmias might reduce myocardial oxygen demand, offering temporary cardioprotection, growing evidence suggests they contribute to a range of cardiovascular abnormalities. Several epidemiological studies have shown a strong association between SDB and the occurrence of bradyarrhythmias [6,25,26]. For this review, bradyarrhythmia is defined by a sinus rate of fewer than 60 beats per minute and sinus pauses longer than 3 s, based on the American Heart Association (AHA), American College of Cardiology (ACC), and Heart Rhythm Society (HRS) guidelines. Additionally, atrioventricular block and conduction disorders are included under the broader categories of sinus node dysfunction and atrioventricular block [27].

The frequency of bradyarrhythmias in sleep apnea has been thoroughly researched, even in the early days of sleep research. Guilleminault and colleagues discovered that 48% of 400 patients with OSA had significant nocturnal arrhythmia, with 18% experiencing bradyarrhythmia [19]. Similarly, another study found that nearly half of the patients with OSA experienced significant nocturnal cardiac arrhythmias, with bradyarrhythmia episodes occurring in 17% to 26% of cases on average [20]. In a study population of 1394 patients, patients with and without SDB had similar baseline risk factors for bradyarrhythmias, such as age, body mass index (BMI), and hypertension (*p* < 0.001). The study revealed that the OSA group had increased rates of Sinus Bradycardia, Sinus Pause (arrest > 2 s), and AV block, highlighting the significant role of SDB as an independent risk factor in the onset of bradyarrhythmias [28].

A meta-analysis further demonstrated that the combined prevalence of daytime and nocturnal bradycardia was 25% (95% CI: 18.6–32.7) and 69.8% (95% CI: 41.7 to 88.2), respectively [7]. Furthermore, bradyarrhythmias, such as sinus bradycardia, sinus pauses, and sinoatrial and atrioventricular block, are the most common cardiac arrhythmias during sleep in patients with SDB, particularly in those exhibiting signs of nocturnal oxygen desaturations [29,30]. Another recent study has found that the prevalence of nocturnal sinus bradycardia in SDB is between 7.2% and 40%, second or third-degree atrioventricular block is between 1.3% and 13.3%, and sinus pauses are between 3.3% and 33% [6,24,27]. Despite this prevalence, most bradyarrhythmias associated with OSA often do not require pacemaker implantation, as these arrhythmias are connected to apneic episodes, over 80% of which occur during REM sleep [27].

## 6. Epidemiological Study Limitations and Considerations

While the studies reviewed provide robust evidence linking SDB to cardiac arrhythmias, several limitations warrant consideration. Many studies, such as DREAM and SHHS, relied on observational designs, which limit their ability to establish causality and are prone to confounding factors despite multivariable adjustments. Furthermore, the variability in diagnostic criteria for SDB and arrhythmias across studies introduces heterogeneity, complicating the comparison and synthesis of results. For instance, differences in AHI thresholds, PSG versus home sleep apnea testing (HSAT) use, and variations in arrhythmia definitions could lead to inconsistent findings. Additionally, the reliance on self-reported measures, such as sleep questionnaires, may result in misclassification bias, particularly in large-scale epidemiological studies. Another critical limitation is the underrepresentation of specific populations, such as women and minority groups, in some of these studies, which limits the generalizability of the findings. These limitations highlight the need for more rigorous and representative studies to fully reveal the complex relationship between SDB and cardiac arrhythmias.

## 7. Shared Risk Factors Between Sleep-Disordered Breathing and Cardiac Arrhythmias

SDB and cardiac arrhythmias share several risk factors that highlight the connection between these two conditions. Emerging research highlights the complex relationship between SDB and cardiac arrhythmias, influenced by comorbidities like hypertension and obesity and demographic factors such as age and ethnicity. Recognizing these shared risk factors is crucial in identifying at-risk patients and developing comprehensive treatment strategies.

Obesity is a complex, multifaceted condition that plays a pivotal role in the pathogenesis of both OSA and cardiovascular diseases. The interplay between obesity and these conditions is mediated by chronic systemic inflammation, autonomic dysregulation, and adverse cardiac remodeling. Increased adiposity, particularly central obesity, is strongly associated with hemodynamic alterations and structural cardiac changes, predisposing individuals to arrhythmogenic conditions [31]. Effective management of obesity, incorporating lifestyle modification, pharmacotherapy, and, where appropriate, bariatric surgery, is critical not only for mitigating the risk of OSA but also for addressing the broader spectrum of obesity-related cardiovascular complications, including arrhythmias [31]. Cardiac rehabilitation’s multidisciplinary approach encompassing tailored physical activity, dietary intervention, and psychosocial support remains a cornerstone in optimizing long-term cardiovascular outcomes in this population [31]. Additionally, the advent of novel pharmacological agents, such as GLP-1 receptor agonists, offers promising adjunctive therapies to enhance weight management and reduce the incidence of obesity-related cardiovascular events.

In a study by Gami et al., the connection between OSA, obesity, and AF was further elucidated by tracking a cohort of 3542 adults over an average follow-up of 4.7 years. Each 1 kg/m^2^ increase in BMI was associated with a 3% rise in AF risk (HR 1.03; *p* < 0.001), and these relationships remained significant after adjusting for age, sex, and other cardiovascular conditions. Importantly, the study revealed that OSA had a stronger association with AF than obesity alone, as indicated by the hazard ratio for AF in individuals with SDB (HR 2.18) compared to those with obesity (HR 1.49) [32]. The findings of Gami et al. reinforce the critical need for integrated management strategies that address obesity and sleep apnea to reduce the incidence of AF and improve cardiovascular outcomes.

May et al. revealed how different indices of SDB predict new-onset AF in a cohort of 843 older men who were initially AF-free. Over a mean follow-up of 6.5 years, 99 participants developed AF, representing 12% of the cohort [33]. The study identified CSA and Cheyne-Stokes respiration (CSR) as significant predictors of AF. In fully adjusted models accounting for age, race, body mass index, heart failure (HF), and OSA severity, CSA (OR: 2.58) and CSR (OR: 2.27) remained strongly linked to incident AF [33]. This association was even more pronounced in men 76 and older, with odds ratios of 9.97 for CSA and 6.31 for CSR [33]. These findings highlight CSA’s importance as an independent risk factor for atrial arrhythmogenesis and emphasize its role in AF risk stratification for older adults. Even after excluding those with a history of HF, the association between CSA and AF remained strong, indicating CSA’s distinct role in AF development.

Race and ethnicity are also significant risk factors for OSA and AF. The Multi-Ethnic Study of Atherosclerosis (MESA) provided insight into these relationships by analyzing over 2200 individuals from diverse racial and ethnic backgrounds. African Americans had significantly higher odds of sleep apnea syndrome (SAS), with an odds ratio of 1.78 [34,35,36]. They also faced a fivefold increase in the risk of short sleep duration (OR = 4.95) and nearly double the risk of excessive daytime sleepiness (OR = 1.89) [34,35,36]. Hispanics were 2.14 times more likely to develop severe SDB (OR = 2.14, 95% CI: 1.40–3.28) and 1.8 times more likely to have a short sleep (OR = 1.80, 95% CI: 1.26–2.58) than Caucasians [34,35,36]. Meanwhile, Chinese Americans were 37% more likely to have severe SDB (OR = 1.37) and 2.31 times more likely to have short sleep (OR = 2.31) [34,35,36]. Despite these elevated risks, fewer than 16% of individuals with moderate to severe SDB were aware of their condition. Underdiagnoses were particularly noticeable among Chinese Americans, where only 7.4% with an AHI of ≥15 reported a prior diagnosis [34,35,36]. Consequently, higher SDB prevalence among these ethnic groups could contribute to elevated AF risk, highlighting the importance of recognizing and addressing undiagnosed sleep apnea as a modifiable risk factor in reducing atrial fibrillation across diverse populations.

Building on these findings, the Reasons for Geographic and Racial Differences in Stroke (REGARDS) trial assessed the association between OSA and AF across a biracial cohort. The study included 20,351 African American and Caucasian adults, with participants stratified into low-risk or high-risk OSA groups based on their responses to the Berlin Sleep Questionnaire [36]. AF was determined through a self-reported history of physician-diagnosed AF or electrocardiogram (ECG) findings. In the overall cohort, the prevalence of AF was significantly higher in the high-risk OSA group compared to the low-risk group (9% vs. 7%; *p* < 0.0001) [36]. Subgroup analysis by race demonstrated that African American participants with high-risk OSA had 58% increased odds of AF (OR = 1.58), while the association was not significant for whites (OR = 1.12) [36]. African American individuals with high-risk OSA are particularly susceptible to AF, which necessitates focused screening and management strategies tailored to this demographic to reduce the burden of AF.

## 8. Pathophysiological Mechanisms Linking SDB to Arrhythmias

SDB is intricately linked to the development of cardiac arrhythmias through intermittent hypoxia, autonomic dysfunction, and dynamic intrathoracic pressure changes.

### 8.1. Role of Intermittent Hypoxia

**Figure 1:**
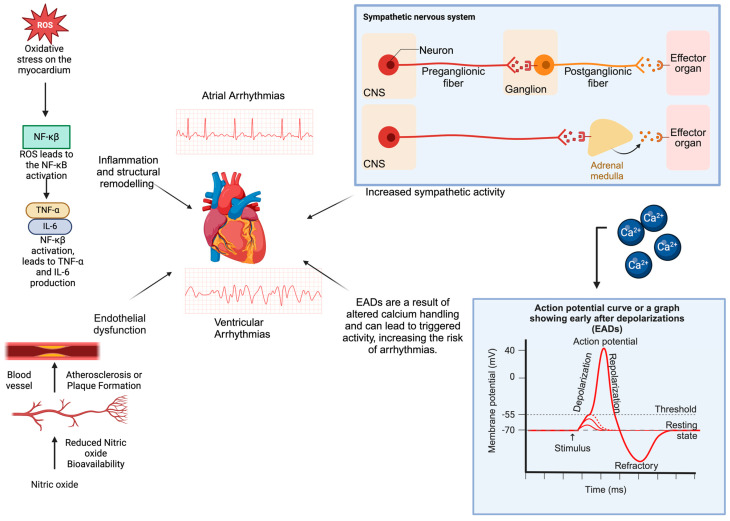
Pathways linking oxidative stress, sympathetic activation, and endothelial dysfunction to cardiac arrhythmias. Oxidative stress on the myocardium triggers NF-κB activation, producing pro-inflammatory cytokines TNF-α and IL-6, which drive inflammation and structural remodeling of the heart. Enhanced sympathetic activity increases the risk of arrhythmias through elevated adrenergic signaling. Endothelial dysfunction, marked by reduced nitric oxide bioavailability, promotes atherosclerosis and plaque formation. Altered calcium handling in cardiomyocytes results in early afterdepolarizations (EADs), further predisposing the heart to arrhythmic events. License: BioRender.com/s95z248.

Intermittent hypoxia, a hallmark of OSA, triggers systemic inflammation by activating transcription factors HIF-1 and NF-κB. These factors promote cytokine production and inflammatory cell activation, leading to vascular changes. NF-κB regulates inflammatory mediators like TNF-α, exacerbating cardiovascular damage by stimulating further release of cytokines, chemokines, and adhesion molecules [37,38]. Meanwhile, HIF-1 extends inflammation by enhancing myeloid cell survival [37,38]. Additionally, Intermittent hypoxia induces oxidative stress, generating reactive oxygen species (ROS), directly damaging the myocardium and activating signaling pathways that amplify cardiac stress via inflammatory mediators. This inflammatory state promotes myocardial structural remodeling, including fibrosis, a key substrate for arrhythmias. It also heightens sympathetic activity, elevating circulating catecholamines. Chronic sympathetic activation alters calcium handling and action potential duration, leading to early afterdepolarizations (EADs) and triggered activity, which are key mechanisms in VA development [39]. It also contributes to endothelial dysfunction and atherosclerosis by reducing nitric oxide (NO) bioavailability and increasing endothelial permeability [39]. This dysfunction accelerates atherosclerosis and coronary artery disease, compromising myocardial perfusion and exacerbating ischemic injury (Figure 1). It also acts as a potent arrhythmogenic trigger that promotes reentrant and non-reentrant arrhythmias through altered conduction velocity and conduction block zones [39]. These structural, autonomic, and electrophysiological changes create a highly arrhythmogenic substrate. Paradoxically, during rem sleep, intermittent hypoxia can stimulate carotid body chemoreceptors, resulting in vagal hyperactivity and increased parasympathetic activity, leading to bradycardias and sinus pauses exceeding 2 s [6,28,40]. Post-apneic hyperventilation triggers tachycardia via sympathetic activation, and the cyclical shifts between sympathetic and parasympathetic activity in OSA may explain the link between brady-tachy syndrome and OSA [29,41,42].

### 8.2. Role of Autonomic Imbalance

**Figure 2:**
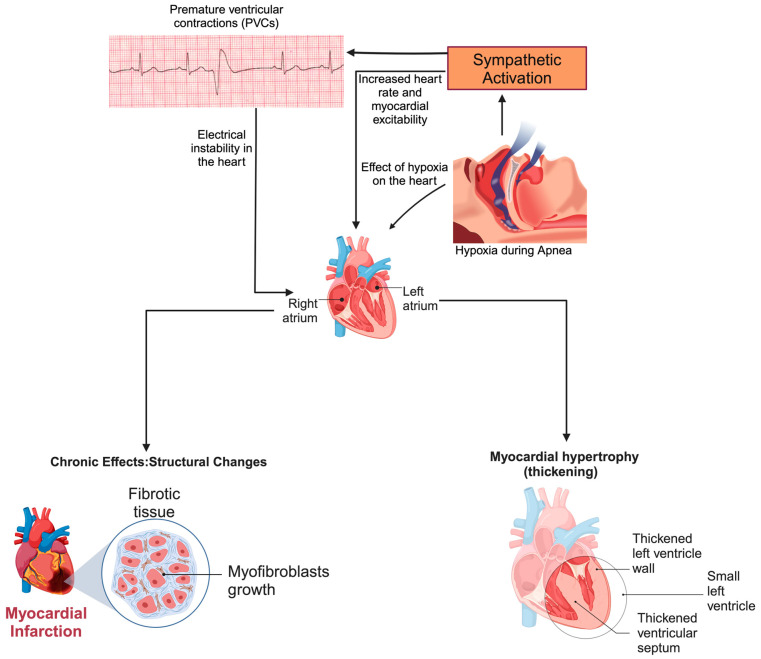
Pathophysiological pathways linking hypoxia and sympathetic activation to cardiac arrhythmias and structural remodeling. Hypoxia during apnea episodes triggers sympathetic activation, increasing heart rate and myocardial excitability. This heightened excitability causes premature ventricular contractions (PVCs) and electrical instability in the heart. Chronic exposure to these conditions results in structural changes such as myocardial hypertrophy and fibrosis. The cumulative effect of hypoxia-induced sympathetic activation promotes the development of an arrhythmogenic substrate. License: BioRender.com/g68j331.

Sleep apnea is key in triggering and worsening AF through mechanisms involving the cardiac autonomic nervous system (ANS). Acidosis, hypoxia, and hypercarbia caused by intermittent hypoxia activate ganglionated plexi (GPI), which contain complex networks of neurons that collectively orchestrate the autonomic response to apnea [43,44,45,46]. Increases in GPI activity result in sympathetic excitation, which results in increased heart rate and systolic blood pressure and parasympathetic bursts periodically that reduce the atrial refractory period [43,44,45,46]. The resulting autonomic imbalance contributes to progressive atrial remodeling, further complicating AF management in OSA patients. Autonomic imbalance in OSA also contributes to the development of VA through the dynamic interplay between the sympathetic and parasympathetic nervous systems. Apnea-induced hypoxia initially enhances parasympathetic activity, leading to bradycardia, followed by a sharp rise in circulating catecholamines, particularly adrenaline, which results in a hypersympathetic response that predisposes individuals to PVCs, which can progress to more severe arrhythmic events (Figure 2) [29,47].

### 8.3. Role of Intrathoracic Pressure Dynamics

The negative intrathoracic pressure generated during inhalation against a blocked airway in OSA significantly disrupts left ventricular (LV) mechanics, impacting diastolic and systolic functions. This pressure increases transmural pressure across the left ventricle, raising afterload and complicating ventricular filling [48,49]. Concurrently, it enhances venous return, leading to right ventricular enlargement, further impairs left ventricular filling by shifting the interventricular septum [48,49]. As a result, there is a marked decline in LV stroke volume, particularly during the diastolic phase, due to decreased LV transmural pressure, reduced integrated mitral flow, and decreased preload [50]. The heightened afterload during systolic phases further complicates LV emptying, emphasizing the intricate relationship between OSA-induced intrathoracic pressure changes and cardiac function [50]. These alterations collectively reduce stroke volume and cardiac output, setting the stage for further complications, including the synergistic effects of diastolic and systolic dysfunction. Furthermore, negative intrathoracic pressure swings during apneic episodes enhance wall stress in the atria and aorta, contributing to nocturnal atrial arrhythmias. These pressure drops stretch cardiac structures, altering ventricular filling dynamics and triggering mechanosensitive ion channels [51]. This stretch-induced electrical instability precipitates premature ventricular contractions (PVCs) and other VA [51]. The rapid ventricular strain also fosters heterogeneous electrical responses, promoting reentrant circuits, which exacerbate VA risk [51]. Evidence indicates that myocardial regions with higher mechanical compliance may act as foci for stretch-activated arrhythmias [51]. Similar to the role of altered intrathoracic pressure dynamics in AF and VA, fluctuating intrathoracic pressures in SDB lead to myocardial stretch and transmural pressure swings, promoting bradyarrhythmias via enhanced parasympathetic activation [6,52]. Over time, unrecognized SDB may drive cardiac biochemical changes and structural and electrical remodeling, potentially through sinus node fibrosis and endothelial abnormalities resulting in bradyarrhythmias [6,53].

This discussion emphasizes the multifaceted and interrelated mechanisms by which SDB, mainly through factors like intermittent hypoxia, oxidative stress, autonomic dysregulation, and mechanical stress, contributes to developing cardiac arrhythmias. Understanding the role of these pathophysiological processes in disrupting cardiac function and structure is vital for advancing therapeutic strategies to mitigate arrhythmic risks in patients with SDB.

## 9. Role of Continuous Airway Pressure Therapy (CPAP) and Non-CPAP Therapies in Reducing Cardiac Arrhythmias in SDB

This section discusses the impact of CPAP therapy on AF, VA, and Bradyarrhythmias in patients with OSA, highlighting the findings from multiple studies.

The meta-analysis “Effect of Obstructive Sleep Apnea Treatment on Atrial Fibrillation Recurrence” examined data from seven prospective cohort studies involving 1087 patients. It revealed a significant reduction in AF recurrence among CPAP users, with a relative risk of 0.58 [54]. This therapeutic benefit was consistent across various patient demographics and clinical backgrounds, including those who underwent pulmonary vein isolation (PVI). Supporting these findings, the systematic review “Meta-Analysis of Continuous Positive Airway Pressure as a Therapy of Atrial Fibrillation in Obstructive Sleep Apnea” by Qureshi et al., which included eight studies with 4516 participants, demonstrated a 44% reduction in AF recurrence rates among OSA patients treated with CPAP (pooled relative risk was 0.56 (*p* < 0.001), with minimal heterogeneity (I^2^ = 0%)) [55].

Additionally, the meta-analysis “Efficacy of Catheter Ablation of Atrial Fibrillation in Patients with Obstructive Sleep Apnoea with and without Continuous Positive Airway Pressure Treatment,” which included five observational studies involving 3743 patients, highlighted that OSA patients had a 31% greater risk of AF recurrence post-catheter ablation compared to non-OSA patients [56]. Notably, OSA patients not undergoing CPAP therapy had a 57% higher recurrence risk. In contrast, those treated with CPAP had recurrence rates similar to non-OSA patients [56].

In the ORBIT-AF trial, the effects of CPAP therapy on AF progression in patients with OSA were evaluated. Among the 10,132 patients enrolled, 1841 had a diagnosis of OSA. These patients exhibited a higher symptom burden and increased hospitalization rates, with 43 events per 100 patient-years compared to 35 events per 100 patient-years in those without OSA (HR:1.12; *p* = 0.0078) [57]. Importantly, CPAP therapy significantly reduced AF progression among OSA patients. The adjusted HR for progression to more persistent forms of AF in CPAP-treated patients was 0.66 (*p* = 0.021) [57]. Despite the lack of significant differences in mortality or major adverse cardiovascular events between OSA and non-OSA patients, the marked reduction in AF progression with CPAP therapy is clinically significant.

In the SAVE study, while AF was not the focus, the findings showed that CPAP therapy did not reduce AF occurrences within the broader scope of cardiovascular outcomes. This large-scale trial included 2717 patients suffering from moderate-to-severe OSA, alongside existing cardiovascular disease, seeking to understand CPAP’s impact on major cardiovascular events. Even though CPAP substantially lowered the apnea-hypopnea index—from 29.0 events per hour at baseline to 3.7 during follow-up, it did not significantly prevent recurrent cardiovascular events, including AF [58]. New-onset AF occurred in 22 patients (1.6%) in the CPAP group versus 15 patients (1.1%) in the usual care group, yielding a hazard ratio of 1.46 (*p* = 0.26), showing no significant reduction in AF with CPAP therapy [58]. However, CPAP did make a positive difference in other areas. It significantly reduced daytime sleepiness, with patients showing an adjusted mean difference of −2.5 on the Epworth Sleepiness Scale (*p* < 0.001), effectively decreasing snoring [58]. Moreover, CPAP users saw improvements in mood and overall health-related quality of life. This was reflected in the enhanced scores on both the anxiety and depression subscales of the Hospital Anxiety and Depression Scale as well as the physical and mental components of the SF-36 health survey [58].

Having established the role of CPAP therapy in reducing AF recurrence, exploring its impact on ventricular arrhythmias in patients with OSA is crucial. CPAP therapy is increasingly recognized for its potential to reduce VA in patients with coexisting HF and OSA. This synthesis of recent studies focuses on the role of CPAP in moderating these cardiac irregularities.

A 2000 study by S. Javaheri focused on the effectiveness of CPAP on HF patients with sleep apnea, particularly on ventricular irritability. This research involved 29 male patients, each diagnosed with substantial sleep-disordered breathing (AHI > 15) episodes per hour. After CPAP treatment, the results varied: 16 patients experienced a significant reduction in AHI and associated symptoms, while 13 saw no notable changes [59]. Among the responders, CPAP markedly improved sleep-disordered breathing, reduced arousal due to breathing disorders, enhanced arterial oxygen saturation, and decreased nocturnal PVCs and couplets [59]. Specifically, PVCs decreased from an average of 66 to 18 occurrences per hour, and couplets dropped from 3.2 to 0.2 per hour [59]. Building on these findings, a 2005 randomized controlled trial explored the effects of CPAP on VE in HF patients with OSA. The study enrolled 18 HF patients with an AHI > 20 per hour and more than ten ventricular premature beats (VPBs) per hour of sleep. These patients were randomized into control and CPAP treatment groups, with medical treatment optimized for all participants. Over one month, the treatment group demonstrated notable improvements: a substantial reduction in AHI (*p* < 0.001), an increase in minimum oxygen saturation (*p* = 0.05), and a 58% reduction in the frequency of VPBs during sleep (from 170 to 70 per hour; *p* = 0.01) [60]. Additionally, urinary norepinephrine concentrations, an indicator of sympathetic nervous system activity, were significantly reduced in the CPAP group [60]. Further corroborating these results, Seyis et al. investigated the effects of CPAP therapy on VA in 80 patients with co-existing OSA and HF, divided into CPAP and control groups. Over six months, comprehensive assessments were conducted, including electrocardiography, 24-h Holter monitoring, echocardiography, and NT-proBNP level measurements [61]. The findings demonstrated a substantial reduction in PVCs and improvements in electrocardiographic indices like T-peak to T-end interval and corrected QT dispersion in the CPAP group compared to controls (*p* < 0.001 for all) [61]. Moreover, a significant decrease in NT-proBNP levels indicated reduced ventricular wall stress in the CPAP-treated patients [61].

The management of non-SDB-induced bradyarrhythmias typically focuses on the management of the identified cause [27]. However, SDB is a known cause of reversible cardiac arrhythmias, including bradyarrhythmias, and thus, CPAP has the potential to reduce the occurrence of these bradyarrhythmias. Teo and colleagues performed a comprehensive meta-analysis, which showed that compared to non-OSA patients, patients with OSA were associated with ten times the risk of daytime sinus nodal bradycardia (OR: 10.04; 95% CI: 1.44–70.16) [7]. In this meta-analysis and other studies, the treatment of OSA with CPAP had not shown consistent statistically significant relative risk reduction in daytime and nocturnal bradycardia. However, the lack of significance could be due to the notable study duration and small sample sizes in the included studies. These findings point to the increased need for future research to examine if CPAP treatment, compared to the placebo arm, has any prognostic significance in SDB-induced bradyarrhythmias. The 2018 ACC/AHA/HRS guideline recommends treating the underlying sleep disorder and reserving pacemaker placement for persistent bradyarrhythmias despite compliance with positive airway pressure treatment [6,7,20,26,62].

The concurrent findings from these studies strongly support the efficacy of CPAP therapy in managing cardiac arrhythmias related to SDB. The consistent improvements in cardiac metrics and reductions in arrhythmias provide a compelling argument for incorporating CPAP as a standard therapeutic measure in this patient population. Future research should aim to confirm these findings through more extensive, multicentric trials to establish a more standardized treatment approach that could potentially enhance these patients’ quality of life and survival rates (Table 2).

Non-CPAP therapeutic interventions for OSA are gaining increasing recognition, particularly for patients with concurrent cardiac arrhythmias. Positional therapy and weight reduction programs have demonstrated effectiveness, especially in cases where OSA severity is influenced by body position or obesity [63]. Pharmacological options, such as atomoxetine and oxybutynin, target pharyngeal dilator muscles to maintain airway patency during sleep, although their modest impact on the apnea-hypopnea index (AHI) and associated side effects, like dry mouth and fatigue, limit long-term adherence [64]. Mandibular Advancement Devices (MADs) have emerged as a primary alternative for mild to moderate symptomatic OSA, offering enhanced patient adherence and a comparable Mean Disease Alleviation rate to CPAP despite challenges such as dental contraindications [63]. Surgical options, including uvulopalatopharyngoplasty (UPPP), maxillomandibular advancement (MMA), and hypoglossal nerve stimulation (HNS), target anatomical obstructions or enhance airway patency through various mechanisms. UPPP enlarges the airway by removing or repositioning soft tissues, MMA surgically advances the jawbone to expand airway space, and HNS employs electrical stimulation to prevent airway collapse [65]. Each of these interventions provides a critical component in the comprehensive management of OSA in patients where CPAP therapy is either insufficient or poorly tolerated.

## 10. Future Directions

Future directions in the management of SDB emphasize personalized medicine, focusing on tailoring treatments to the unique characteristics of each patient. Given the strong association between SDB and cardiovascular conditions like cardiac arrhythmias, addressing the heterogeneity of these manifestations is essential for improving outcomes and reducing cardiovascular risks. By considering genetic background, lifestyle, and comorbidities like hypertension, diabetes, and other factors, clinicians can identify specific phenotypes and match them with the most appropriate interventions. Although CPAP remains the standard treatment, patient compliance often limits its effectiveness. Innovations such as smart CPAP devices, incorporating advanced algorithms and patient feedback, aim to enhance adherence by offering a more personalized experience. Additionally, tailored hypoglossal nerve stimulators and ASV provide customized solutions for those unresponsive to traditional treatments. The integration of telemedicine and wearable technologies offers the potential for continuous monitoring and early intervention, further individualizing care and potentially improving long-term clinical outcomes. Understanding the molecular mechanisms underlying OSA, particularly the roles of hypoxia-inducible factors, inflammatory cytokines, and oxidative stress, is crucial for advancing these strategies. Moreover, cutting-edge approaches like gene editing and regenerative medicine hold promise for correcting genetic anomalies and mitigating cardiac remodeling, which could have profound implications for patient prognosis. To validate these novel therapies and fully realize the potential of personalized medicine, large-scale clinical trials are necessary, which could significantly reshape the therapeutic landscape for OSA and its cardiovascular complications.

## 11. Conclusions

OSA and CSA, the primary forms of SDB, significantly contribute to cardiac arrhythmias. Mechanisms such as intermittent hypoxia, autonomic dysfunction, and intrathoracic pressure changes are central to this relationship. Strong evidence shows that severe SDB substantially increases arrhythmia risk, while CPAP therapy effectively reduces this risk. Early identification and management of SDB are crucial for mitigating cardiovascular risk and enhancing patient outcomes. Future research should aim to refine therapeutic approaches, incorporate personalized medicine, and utilize advanced technologies to address the cardiovascular consequences of SDB better. Emerging technologies in molecular genetics and artificial intelligence (AI) are poised to revolutionize the early detection and management of these conditions. AI-driven diagnostic tools can facilitate early identification of SDB and its cardiac complications, enabling timely and precise clinical interventions.

## Figures and Tables

**Table 1:** Epidemiological studies on cardiac arrhythmias: this table outlines key findings from epidemiological studies investigating the relationship between sleep-disordered breathing (SDB) and atrial fibrillation (AF), ventricular arrhythmia (VA), and sudden cardiac death (SCD).

Study	Population	Outcomes	Key Findings
Sleep Apnea and Atrial Fibrillation Study (2021) [8]	1000 Patients	Prevalence and association of OSA with AF	OSA prevalence ranged from 21% to 74% in AF patients compared to 3% to 49% in controls. The study highlighted the close relationship between OSA and AF.
VARIOSA-AF Study (2019) [11]	72 Patients	Temporal dynamics between SDB and AF	Utilized implanted monitoring devices to track RDI and AF burden. Found a robust correlation between severe nocturnal SDB episodes and increased risk of AF events. Nights with the highest RDI quartile showed a significantly increased risk of AF episodes the following day, with odds ratios up to 10.2 for episodes exceeding 12 h.
Association of Short Sleep Duration and Atrial Fibrillation (2019) [12]	31,079 Patients	Link between sleep duration and AF	For every one-hour reduction in total sleep time, there was a 17% increase in the risk of prevalent AF and a 9% increase in the risk of incident AF. Patients who slept less than 3 h had 2.10 times higher odds of prevalent AF than those who slept more than 6 h.
Healthy Sleep Patterns and Risk of Incident Arrhythmias Study (2021) [13]	403,187 Patients	Impact of sleep quality on AF	Participants with the highest adherence to healthy sleep behaviors showed a 29% reduction in the risk of developing AF. Optimal sleep duration (7–8 h per night) and absence of frequent insomnia were associated with significant reductions in AF risk.
I-STOP-AFib Tria (2022) [14,15]	446 Patients	Effect of sleep reduction on AF	The trial involved 446 participants with paroxysmal AF who tested self-selected triggers, including reduced sleep. Results did not conclusively link reduced sleep to increased AF episodes, suggesting that the impact of sleep disturbances on AF may vary significantly among individuals.
Obstructive Sleep Apnea and the Risk of Sudden Cardiac Death: A Longitudinal Study of 10,701 Adults (2013) [16]	10,701 Patients	Risk of SCD associated with OSA	Severe nocturnal hypoxemia (AHI > 20 and lowest nocturnal oxygen saturation below 78%) significantly predicted SCD, with hazard ratios of 1.60 and 1.81.
CESAAR Study (2019) [17]	202 Patients	Association between SDB and NSVT post-MI	Found that an AHI > 23 was associated with a threefold increase in the risk of NSVT during the subacute recovery phase of acute myocardial infarction.
Disrupted Day-Night Pattern Study (2005) [18]	112 Patients	Increased risk of SCD associated with OSA	Analysis of 112 subjects revealed that 46% of those with OSA died from SCD between midnight and 6 a.m., compared to 21% without OSA (*p* = 0.01). The relative risk of SCD during this period was 2.57 for OSA patients, highlighting the increased nocturnal risk.
Cardiac Arrhythmia and Conduction Disturbances During Sleep in Patients With Sleep Apnea Syndrome (1983) [19]	400 Patients	Prevalence of bradyarrhythmias in OSA patients	Analysis of 400 patients with OSA; 48% had significant nocturnal arrhythmia, with 18% experiencing bradyarrhythmia.
Severe bradyarrhythmias in patients with sleep apnoea (2004) [20]	1394 Patients		Approximately half of the patients with OSA experienced severe nocturnal cardiac rhythm disturbances, with bradyarrhythmia episodes occurring between 17% to 26%.

**Table 2:** Impact of CPAP on atrial fibrillation, ventricular arrhythmias, and bradyarrhythmias. This table presents key findings from studies examining the effect of continuous positive airway pressure (CPAP) therapy on the recurrence of atrial fibrillation (AF) and ventricular arrhythmias in patients with sleep-disordered breathing (SDB).

Study	Population	Key Findings
Effect of Obstructive Sleep Apnea Treatment on Atrial Fibrillation Recurrence (2015) [56]	1087 Patients	The meta-analysis reviewed seven prospective cohort studies involving 1087 patients. CPAP therapy significantly reduced AF recurrence among OSA patients, with a relative risk reduction of 42% (RR 0.58). This benefit was consistent across various patient demographics and clinical backgrounds, including those who underwent pulmonary vein isolation (PVI).
Meta-Analysis of Continuous Positive Airway Pressure as a Therapy of Atrial Fibrillation in Obstructive Sleep Apnea (2015) [57]	4516 Patients	CPAP therapy was associated with a 44% reduction in AF recurrence rates (pooled relative risk: 0.56, *p* < 0.001).
Efficacy of Catheter Ablation of Atrial Fibrillation in Patients with Obstructive Sleep Apnoea with and without Continuous Positive Airway Pressure Treatment (2014) [58]	3743 Patients	CPAP-treated patients had similar AF recurrence rates to non-OSA patients, while non-CPAP-treated OSA patients had a 57% higher recurrence risk in post-catheter ablation.
ORBIT-AF trial (2015) [59]	10,132 Patients	CPAP therapy was associated with a significant reduction in AF progression (adjusted HR: 0.66, *p* = 0.021) and decreased hospitalization rates in OSA patients.
SAVE study (2016) [60]	2717 Patients	CPAP therapy did not significantly reduce AF occurrences (HR: 1.46, *p* = 0.26) but significantly improved daytime sleepiness, reduced snoring, and enhanced overall quality of life.
Effects of Continuous Positive Airway Pressure on Sleep Apnea and Ventricular Irritability in Patients With Heart Failure (2000) [61]	29 Patients	CPAP therapy significantly reduced AHI, increased arterial oxygen saturation, and decreased the frequency of nocturnal PVCs from an average of 66 to 18 per hour and couplets from 3.2 to 0.2 per hour.
Effect of continuous positive airway pressure on ventricular ectopy in heart failure patients with obstructive sleep apnoea (2005) [62]	18 Patients	CPAP therapy led to a substantial reduction in AHI, increased minimum oxygen saturation, decreased the frequency of VPBs by 58% (from 170 to 70 per hour), and reduced urinary norepinephrine levels.
The Effects of Continuous Positive Airway Pressure on Premature Ventricular Contractions and Ventricular Wall Stress in Patients with Heart Failure and Sleep Apnea (2018) [63]	80 Patients	CPAP therapy significantly reduced the incidence of premature ventricular contractions, improved electrocardiographic indices (T-peak to T-end interval, corrected QT dispersion), and decreased NT-proBNP levels.
Efficacy of continuous positive airway pressure on arrhythmias in obstructive sleep apnea patients (2022) [37]	1394 Patients	CPAP significantly reduced the occurrence of sinus bradycardia (*p* = 0.001) and sinus pause (*p* = 0.004).
